# Effects of “Taking the Waist as the Axis” Therapy on trunk postural control disorder after stroke: A randomized controlled trial

**DOI:** 10.3389/fnagi.2023.1040277

**Published:** 2023-01-27

**Authors:** Rong Cui, Hongtao Liu, Meng Li, Jie Wang, Junjie Mao, Weidong Ni, Furong Wang, Jingxian Pan, Long Yu, Yan Wang, Yanmin Wang, Pufeng Huang, Gaiyan Li, Yi Zhao, Ning Zhu, Chen Chen, Ziyang Pan, Ying Zhang, Weijie Fu, Jianzhong Yang

**Affiliations:** ^1^Department of Rehabilitation, Shanghai Xuhui Central Hospital, Shanghai, China; ^2^School of Exercise and Health, Shanghai University of Sport, Shanghai, China; ^3^Department of Respiratory and Critical Care Medicine, Shanghai Xuhui Central Hospital, Shanghai, China; ^4^Department of Neurology, Shanghai Xuhui Central Hospital, Shanghai, China; ^5^Shanghai Hongrun Boyuan School, Shanghai, China; ^6^Shanghai Yichuan Middle School, Shanghai, China

**Keywords:** Tai Chi, “Taking the Waist as the Axis” Therapy (WAT), trunk postural control disorder, selective activity, stroke

## Abstract

**Background:**

Sufficient attention to trunk rehabilitation after stroke is still lacking. Loss of trunk selective activity is considered to be the leading cause of trunk postural control disorder after stroke. “Taking the Waist as the Axis” Therapy (WAT) was developed as a combination of the concept of “Taking the Waist as the Axis” from Tai Chi and the rehabilitation of trunk dysfunction after stroke. The present clinical trial examined and assessed the effects of WAT on stroke patients.

**Methods:**

A total of 43 stroke hemiplegic patients with trunk postural control disorder, whose Trunk Impairment Scale (TIS) scoring between 8 and 18, participated in the present study and were allocated randomly to the experimental (*n* = 23) or control groups (*n* = 20). The experimental group received WAT plus conventional therapy, and the control group received “Trunk Selective Activity” Therapy (TSAT) plus conventional therapy. Both groups received treatment once daily and 5 times per week for 3 weeks. The Trunk Impairment Scale (TIS), Fugl-Meyer Assessment (FMA), Berg Balance Scale (BBS), change of Intra-abdominal Pressure (IAP), static balance ability assessment, rapid ventilation lung function test and the Modified Barthel Index (MBI) were evaluated before and after intervention for both groups.

**Results:**

The experimental group was superior to the control group in TIS [4 (2, 5) vs. 3 (1.25, 4), *p* = 0.030], change of IAP [−3 (−8, −1.33) vs. −0.02 (−3.08, 6), *p* = 0.011], FMA-upper extremity [10 (6, 18) vs. 1 (0, 3), *p* = 0.002], FMA-lower extremity [2 (1, 4) vs. 1 (0, 2), *p* = 0.009] and FMA [14 (7, 21) vs. 2 (0.25, 3.75), *p* = 0.001]. Within experimental group, forced vital capacity (FVC) [81.35 (63.30, 94.88) vs. 91.75 (79.40, 97.90), *p* = 0.02] was significantly improved.

**Conclusion:**

WAT was an effective trunk treatment after stroke, which significantly improved the patients’ trunk posture control ability, motor function and forced vital capacity. However, the results still need to be interpreted with caution for the intervention only lasted for 3 weeks.

## Introduction

Trunk postural control disorder is a consequence of hemiplegia after stroke, and has been closely associated with impaired balance, mobility and functional independence (Haruyama et al., 2017). It is characterized by problems with rigid movement, abnormal muscle tone and weight-bearing asymmetry (Brown et al., 1997; Huang et al., 2013; Jamal et al., 2018). Compared with healthy people, stroke patients usually show greater trunk postural oscillations and altered muscular activation, which would increase the risk of falling during walking and standing (De Luca et al., 2020). In addition, abnormal elevation of the thorax and the decreased activity of diaphragm due to impaired trunk postural control, which in turn impairs lung function (Laroche et al., 1988). The main factor contributing to posture disorder is the loss of trunk selective activity, especially the loss of trunk flexion, lateral flexion and rotation. For example, when patients bent their trunk laterally, they could not keep the trunk extended synchronously. In addition, the trunk and limbs could not move independently, such as sitting from the supine position with lower limb flexion, standing with the trunk tilted back and the hips extended, walking through pelvic lifting to complete a lower limb stride, moving the hemiplegic upper limb with hyperextension of the spine, etc. (Davies, 1990). Trunk performance could also predict the functional status and prognosis after stroke (Souza et al., 2019). It was proved that the initial ability of trunk postural control after stroke could predict the performance of activity of daily living (ADL) after 6 months (Hsieh et al., 2002). To date, more and more attention has been paid to the rehabilitation of hemiplegic limbs, while the ability of trunk selective movement has been largely ignored, yet it is critical to the recovery of motor functions in hemiplegic patients after stroke (Saeys et al., 2012).

At the present time, the main intervention for selective trunk activity dysfunction after stroke, such as “Trunk Selective Activity” therapy (TSAT), was designed based on the Bobath concept and emphasizes the regulation of trunk selective activity and the integration of postural control, as well as the task performance for developing coordinated movement (Huseyinsinoglu et al., 2012). Trunk treatments that focus on the intensive training of trunk flexion, extension and lateral flexion can positively influent trunk performance, balance and the walking ability of a stroke patient (Brock et al., 2011; Kılınç et al., 2016). Besides, our daily activities contain a variety of trunk rotation, which means that training focused on trunk rotation is warranted in trunk rehabilitation. However, it has not received enough attention in TSAT. Intensive trunk rotation exercises can activate the abdominal muscles, relieve trunk spasticity and improve trunk stability and flexibility (Ng et al., 2001; Niewiadomy et al., 2021). Thus, training focusing on trunk rotation will likely be a promising strategy to restore trunk capacity in hemiplegic patients after stroke.

Tai Chi, a traditional Chinese fitness regimen, has long been employed in translation medicine. Significant improvements have been demonstrated in balance, motor functions and the gait ability of stroke patients after Tai Chi practice (Au-Yeung et al., 2009; Chen et al., 2015; Kim et al., 2015; Li et al., 2018). Most patients with mild to moderate motor dysfunctions were recruited and taught in a group mode under the guidance of experienced Tai Chi coaches in previous studies (Li et al., 2012; Bhalsing et al., 2018). However, it is difficult for patients with moderate to severe motor dysfunctions to practice Tai Chi movements as normal practitioners, for example due to the increased risk of falls or other injuries (Zhao et al., 2021, 2022). Accordingly, Tai Chi practice should be refined and documented to form a set of practical and scientific rehabilitation programs according to the specific dysfunctions of individual stroke survivor.

The “Taking the Waist as the Axis” is an essential concept of Tai Chi and is firmly ingrained throughout its entire practice. The waist is a vital part of the trunk, which helps with limb movements (Fu and Swaim, 1999). Tai Chi develops flexibility through various circular or arc-shaped movements of the waist, promoting motion of the limbs. Tai Chi theory often mentions: “Dominate in the waist” and “Always pay attention to the waist” (Ma, 2006). We applied these principles to stroke patients, essentially “teaching them how to use the trunk flexibly.” With repeated intensive training, the patient learns to use the trunk properly, finally getting rid of themselves of arduous movement patterns. From extensive clinical practice, the investigators chose 8 postures from the 24-form Tai Chi and reformed and summarized them into “Taking the Waist as the Axis” Therapy (WAT). The therapy emphasizes strengthening axial rotation, compound rotation and diagonal rotation of the trunk, and facilitates a one-to-one training mode in sitting or standing positions. Considering that training at least 3 days a week and a training duration of 20–60 min per session are the recommended training intensity, we took a training intensity of 50 min 5 times per week in our study (Billinger et al., 2014).

The purpose of the present trial was to compare the clinical effects of WAT based on Tai Chi, and TSAT based on the Bobath concept in the hemiplegic patient with trunk postural control disorder. We hypothesized that WAT would show enhanced clinical outcomes compared with TSAT on trunk postural control ability, motor functions, balance, lung function and the ability of ADL in hemiplegic stroke patients.

## Methods

### Study design

A single-center, parallel-group, randomized control trial was designed to explore the efficacy of WAT for the treatment of trunk postural control disorders in stroke patients with hemiplegia. The trial design was approved by Shanghai Xuhui Central Hospital Ethics Committee (Approval No. 2021-013) and was registered with the Chinese Clinical Trials Registry Platform (ChiCTR2100043760).

### Participants

All patients were recruited from February 2021 to March 2022 from a cohort of inpatients admitted to the Department of Rehabilitation of Shanghai Xuhui Central Hospital, Shanghai as a result of stroke. Patients were recruited to the trial according to the following criteria:

(1) Physicians screened potential patients and contacted the lead trial researcher;

(2) The researcher introduced the trial concept to potential patients and asked them about their willingness to participate;

(3) The eligibility of patients was assessed;

(4) The enrolled patients provided written informed consent before commencement of the trial.

### The inclusion criteria

(1) First-time stroke

(2) 2 weeks to 6 months after stroke;

(3) Age of 60 to 80 years;

(4) The Trunk Impairment Scale (TIS) scoring 8–18;

(5) The level of standing balance ≥ I;

(6) Unilateral limb dysfunction;

(7) Ability to tolerate at least 40 min exercise and agree to sign the written informed consents.

### The exclusion criteria

(1) An inability of patients to finish a 40 min course of exercise;

(2) A Mini-Mental State Examination (MMSE) score ≤ 23;

(3) Acute diseases of the heart, brain, kidney and other organs.

Patients were randomly allocated to the experimental group or control group according to a computer-based randomized sequence. Before the experiment, sealed opaque envelopes were sent to the patients to determine which group they would be assigned. The specified researcher was responsible for the data collection and group allocation. All patients were evaluated by the specific evaluators who were not involved in the randomization or implementation of interventions. Information exchange was not permitted among researchers during the progress of the research, nor was information collected from the involved patients.

### Intervention

Both groups received conventional rehabilitation therapy which was conducted according to well defined patient daily rehabilitation therapy regimes, including dynamic sitting and standing transition training, proprioceptive training, occupational therapy and balance bar feedback training. The experimental group received WAT based on Tai Chi and the control group received TSAT based on the Bobath concept.

The intervention time in each group was 50 min for each session, with either WAT or TSAT lasting 30 min, followed by conventional rehabilitation therapy for 20 min, conducted 5 times per week for 3 weeks.

### Experimental group

**Figure 1:**
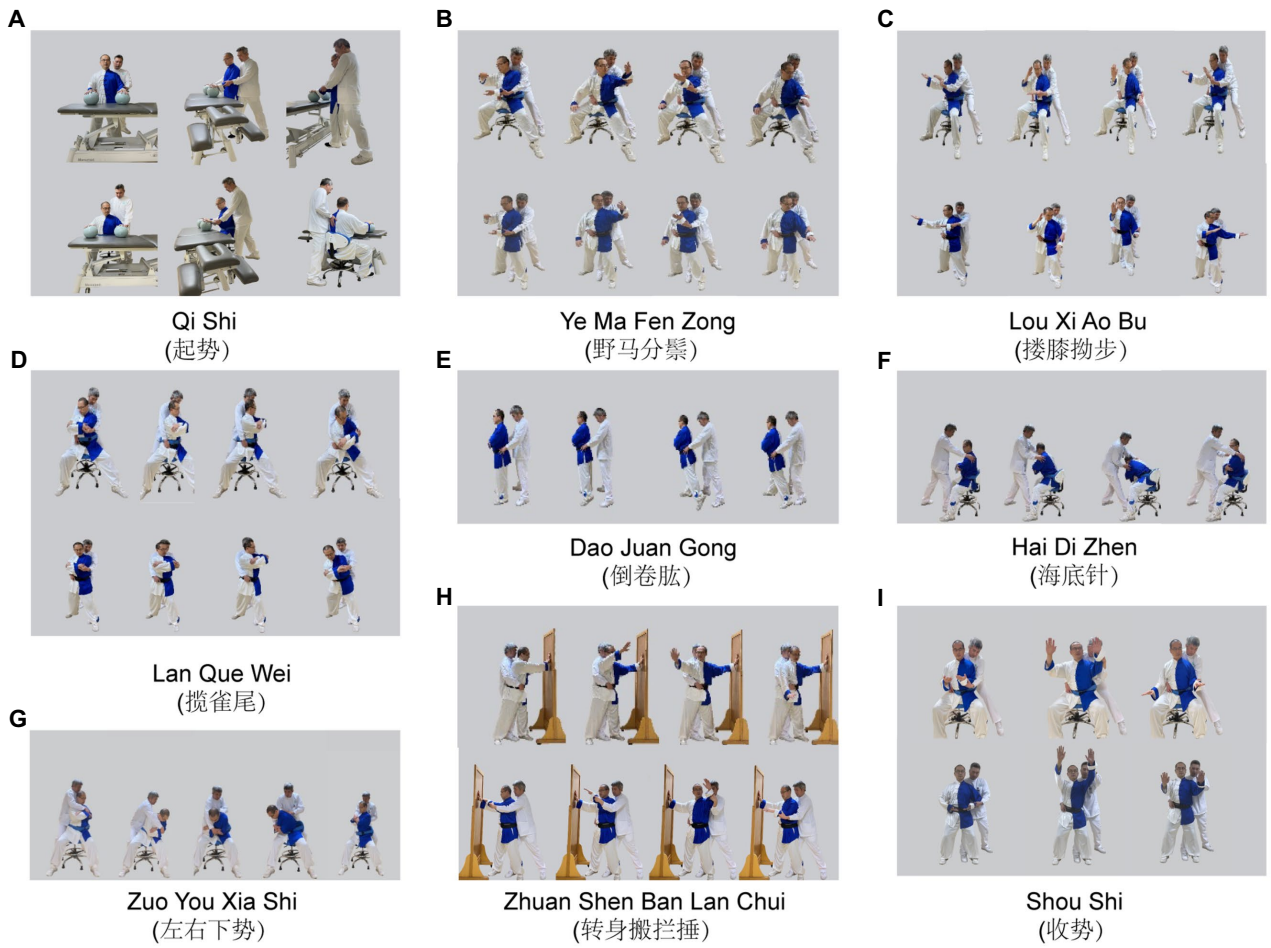
Experimental group (WAT). Two position are applied in the WAT. (1) Sitting position: The patient sits on the swivel chair with fixed chassis with a protective belt or regular chair. Legs slightly wider than shoulders apart. The toe is in line with knee joint. Hold body erect and gaze attending to the main hand. (2) Standing position: lunge position (left): The patient stands with two feet shoulder-width apart, then the right foot externally rotates at 45⁰, weight shift to the right, left foot takes a step forward with the knee facing forward. The therapist stands behind the patient, placing his knee against the popliteal fossa on the affected side (left) and assisting the patient to stand in a lunge with a slight bent knee.

The therapy was carried out by two therapists with formal training in Tai Chi, who had also received 6-month training sessions of WAT. The therapy, involving eight movements, was based on the 24-form Tai Chi that published by the General Administration of Sport of China and the book *Taijiquan “Taking the Waist as the Axis” Hemiplegia Trunk Rehabilitation Manual* (Fu and Swaim, 1999; Liu et al., 2022). The details of the WAT are as follows:

(1) **Qi Shi (起势)**: The patient bends the knees slowly, presses the ball with palms, then pulls it back beside the hips, and inhales simultaneously. Next, the patient stands up slowly, raises arms to shoulder level, and inhales. The therapist locates on the effected side of the patient and assists the patient by controlling the belt and hemiplegic upper limb. The therapist controls the flexion and extension of the patient’s hemiplegic knee with his own knee (Figure 1A).

(2) **Ye Ma Fen Zong** (野马分鬃): The therapist stands behind the patient with his hands control the patient’s hemiplegic arm and waist belt. Under the guidance of the therapist, the patient rotates the trunk (45°–20°–20°–45°) with the weight shifting between two legs. On this basis, the therapist can assist the patient with arm movement (Fen Shou) by controlling the patient’s proximal or distal upper extremity (Figure 1B).

(3) **Lou Xi Ao Bu (搂膝拗步)**: The patient rotates the right arm to the ear, rotates the trunk axially and pushs the palm forward, then turns the left arm inward and rotates it internally. Repeated “Shang Tui Zhang” and “Xia Lou Xi” can be practiced alone (Figure 1C).

(4) **Lan Que Wei (揽雀尾)**: For patients with poor limb function, their arms can be wrapped around the chest. Then, the patient rotates the trunk 45° to the left (Peng), continues to rotate the left 20° more, and then pulls the trunk back with composite rotation (Lv). While for those with better upper extremity function, this can be accomplished with the help provided by the therapist for their upper extremity (Figure 1D).

(5) **Dao Juan Gong (倒卷肱)**: The therapist stands behind the patient and controls the patient’s iliac spine with both hands. The patient rotates his trunk to the right then shifts the weight to the right leg. The therapist applied slight pressure on the patient’s left iliac spine to guide backward extension of the ipsilateral lower limb. Pause for several seconds to stretch the trunk, then continue the contralateral movement (Figure 1E).

(6) **Zuo You Xia Shi (左右下势)**: The therapist stands behind the patient with his hands controlling the patient’s shoulder or controlling patient’s manubrium sternum and thoracic vertebrae. The therapist assists the patient to bend the trunk laterally then to rotate the trunk from left to right, and to stretch the trunk for several seconds. Afterwards, rotate the trunk from right to the starting position and then stretch briefly (Figure 1F).

(7) **Hai Di Zhen (海底针)**: The therapist stands in front of the patient with his hands control the patient’s hemiplegic scapula and arm. With the help of the therapist, the patient bends the trunk and pulls it toward the contralateral toes to accomplish the diagonal rotation movement of the trunk (Figure 1G).

(8) **Zhuan Shen Ban Lan Chui (转身搬拦捶)**: The therapist stands behind the patient with his hands control the hemiplegic hand and waist belt. The patient stands with feet separated and abducent, and the hemiplegic side arm supporting the wall. Rotate the trunk to the contralateral side, the non-hemiplegic arm synchronizes with an arc motion overhead and then extends horizontally to the contralateral side (Figure 1H).

(9) **Shou Shi (收势)**: Feet apart, palms up and out in an arcing motion and press down the hands (Figure 1I).

The control of the trunk rotation was realized by controlling the patient’s thoracic and manubrium sternum and shoulders. Patients gradually superimpose limb movements based on the completion of trunk movements, which can be done with the assistance of a therapist, in addition, special attention needs to be paid to the control of the proximal upper limb and scapular girdle. All the movements above follow slow, continuous, relaxed and repetitive modes and the eyes follow the main hand. The sitting or standing position training is carried out according to a patient’s balance function, with the therapist’s one-to-one instruction or assistance. And a custom-made swivel chair with a fixed chassis or regular chair is used for sitting position training. It is important to note that the trunk and limbs on the non-hemiplegic side should also be exercised accordingly and efforts should be made to alternate the movements of the limbs bilaterally.

### Control group

This therapy consists of training in four different positions and two professional therapists who are familiar with TSAT were in charge of this group. The TSAT is based on the book named *Right in the middle—selective trunk activity in the treatment of adult hemiplegia*, which is based on the Bobath concept (Davies, 1990).

#### 1. Sitting activities

**Figure 2:**
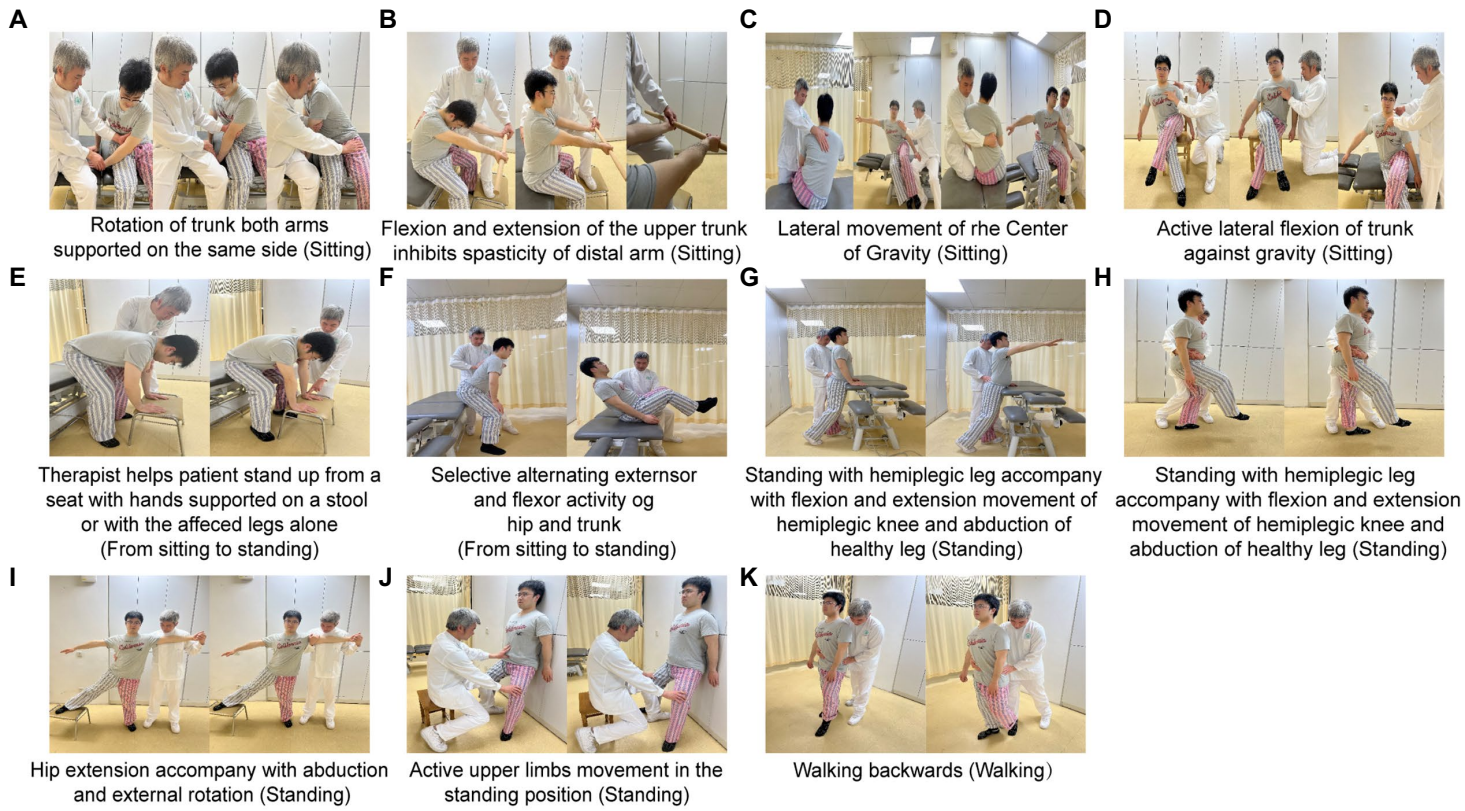
Control group (TSAT). Bobath training consists of four activities: sitting **(A–D)**; sitting to standing **(E,F)**; standing **(G–I)**: and walking **(K)**. Each activity includes selectively exercises the truck and limbs.

(1) Selective flexion and extension movements of the lower trunk.

(2) Rotation of trunk accompanied by flexion movement.

(3) Rotation of trunk with both arms supported on the same side (Figure 2A).

(4) Flexion and extension of the upper trunk which inhibits spasticity of the distal arm (Figure 2B).

(5) Lateral movement about the center of gravity (Figure 2C).

(6) Active lateral flexion of the trunk against gravity (Figure 2D).

#### 2. From sitting to standing

(1) Therapist helps patient stand up from a seat with hands supported on a stool or with the affected leg alone (Figure 2E).

(2) Selective alternating extensor and flexor activity of the hip and trunk (Figure 2F).

#### 3. Standing activities

(1) Anterior and posterior tilt of the pelvis.

(2) Standing with the hemiplegic leg accompanying adduction and abduction of the healthy hip.

(3) Bending trunk forwards and backwards to upright (Figure 2G).

(4) Standing with the hemiplegic leg accompanied by flexion and extension movements of the hemiplegic knee and abduction of the healthy leg (Figure 2H).

(5) Hip extension accompanied by abduction and external rotation (Figure 2I).

(6) Active upper limb movements in the standing position (Figure 2J).

#### 4. Walking

(1) Stabilizing patient’s trunk or helping stretch the hip or supporting the hemiplegic upper limb when walking forward.

(2) Walking backwards, walking to the affected side and walking to the sound side (Figure 2K).

The intervention time in each group was 50 min for each session, with either WAT or TSAT lasting 30 min, followed by conventional rehabilitation training for 20 min, conducted 5 times per week for 3 weeks.

### Primary outcome measures

#### The trunk impairment scale

The TIS consists of 3 components, namely sit-static, sit-dynamic and coordinated assessment. TIS was used to evaluate the ability to keep the trunk stable and to conduct selective activity, including maintaining trunk balance with two legs crossed in the sitting position, trunk lateral bending and rotation of the upper and lower trunk. The scale was scored out of 23, with higher scores indicating better trunk control (Verheyden et al., 2004). The test–retest (ICC = 0.87–0.96) and inter-rater reliability (ICC = 0.87–0.96) of TIS were found to be good (Sorrentino et al., 2018).

### Secondary outcome measures

#### Fugl-Meyer assessment

The FMA, which is a tool to evaluate motor recovery after stroke, is comprised of upper extremity and lower extremity subscales. The maximum score is 100 points, of which 66 points are assigned to the upper extremity (UE) and 34 points to the lower extremity (LE) subscales. A higher score indicates better motor function recovery. Previous studies have shown that the inter-rater reliability of the FMA’s total score (ICC = 0.96), upper extremity motor sub-score (ICC = 0.97) and lower extremity motor sub-score (ICC = 0.92) were high (Gladstone et al., 2002).

#### Berg balance scale

The BBS is a measurement scale of functional balance for stroke population and consists of 14 items. Each item is scored on a scale of 0 to 4 out of 56, with higher scores indicating a better balance function (Downs et al., 2013). The scale has great reliability (ICC = 0.95–0.98) (Blum and Korner-Bitensky, 2008).

#### Change of intra-abdominal pressure

Change of intra-abdominal pressure (IAP), measured by a pressure feedback unit (STABILIZER^™^ Pressure Bio-Feedback, America), was used to evaluate transversus abdominis muscle (TrA) recruitment. The methods have been previously described (Zheng et al., 2021). The smaller change in IAP indicate better control ability of the trunk. A moderate to excellent intra-rater reliability (ICC = 0.5–0.81), inter-rater reliability (ICC = 0.47–0.82) and the correlation values assessing validity have been examined by previous studies (ICC = 0.48–0.90) (Hodges et al., 1996; von Garnier et al., 2009; de Paula Lima et al., 2011).

#### Static balance ability assessment

The static balance abilities of sitting and standing were evaluated using the Prokin proprioception evaluation and training system (PK254P; TecnoBody, Italy). The operational details have been previously published (Wang et al., 2020). The central of pressure (COP) trajectory (mm) and COP area (mm^2^) in sitting and standing position were collected with eyes open and closed, with lower values indicating better balance.

#### Rapid ventilation lung function test

A spirometer was used to perform the rapid ventilation lung function test (X1; XEEK, China). The maximum inspiratory pressure (MIP) and maximum expiratory pressure (MEP) were employed to measure respiratory muscle strength. The forced expiratory volume in the first second (FEV1), forced vital capacity (FVC), peak expiratory flow (PEF), and maximum expiratory mid-flow (MMEF) were used to assess lung ventilation performance. Patients were requested to complete a maximal inspiration and expiration using a mouthpiece linked to a spirometer while seated, and the parameters (% pred) were collected (Zheng et al., 2021).

#### Modified Barthel Index

The Modified Barthel Index (MBI) was developed to assess ADL performance. The index is comprised of 10 items with a maximum score of 100 and is evaluated according to a patient’s self-reporting. A higher score indicates a better ability to perform ADL (Yang et al., 2022).

### Statistical analysis

IBM SPSS Statistics ver. 26.0 software was used for all analyses. General descriptions were used for demographic and clinical characteristics, such as the number of cases and median (IQR). TIS scores are discontinuous variables and presented as medians (IQR). FMA, BBS, MBI, change of IAP, static balance ability, MIP, MEP, FVC, FEV1, PEF and MMEF did not comply with a normal distribution or homogeneity of variance and are presented as medians (IQR). Data were analyzed according to intention-to-treat. The Wilcoxon sign-rank test was used to compare pre- and post-treatment within a group and the Mann–Whitney test to compare pre- and post-treatment differences between groups. A value of *p* < 0.05 was deemed to be statistically significant.

## Results

### Baseline characteristics

**Figure 3:**
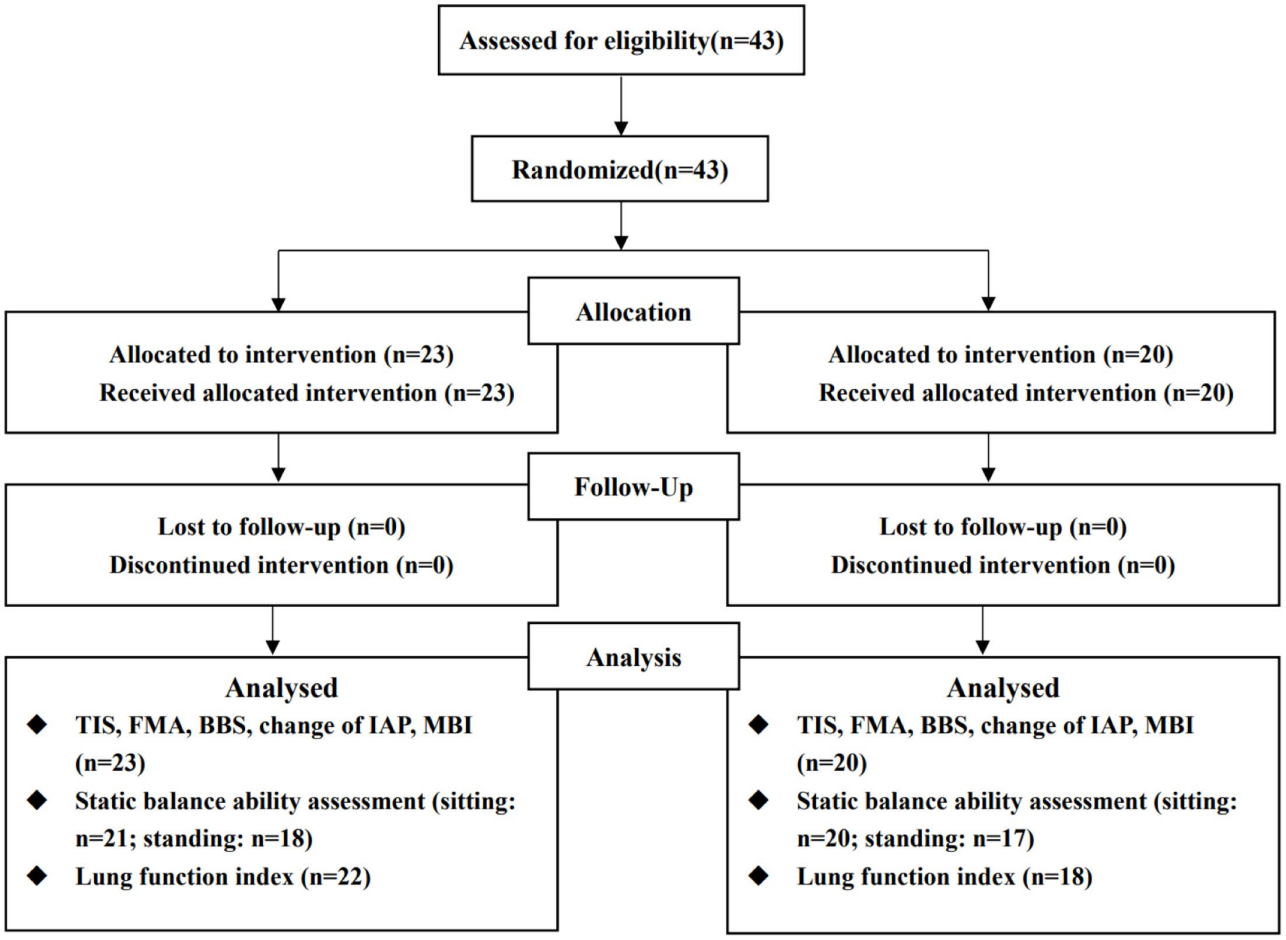
Flow chart. The reason for the missing number of measurement indexes is that some subject could not complete the corresponding evaluation content, according to the unified evaluation standards, due to their functional limitation

Total 43 patients met the inclusion criteria and all completed the study (experimental group, *n* = 23; control group, *n* = 20). Figure 3 shows the flowchart of the trail. No between-group differences in baseline characteristics were found (Table 1) and no serious adverse events occurred.

**Table 1:** Participant characteristics at baseline.

Variables	Experimental group (*n* = 23)	Control group (*n* = 20)	value of *p*
Age (y), Median (IQR)	66 (60, 72)	65.5 (63, 71)	0.922^a^
Course disease (d), Median (IQR)	39 (21, 86)	74.50 (45, 128.75)	0.111^a^
Sex, n (%)			
Male	18 (78)	17 (85)	0.862^b^
Female	5 (22)	3 (15)	
Affected body side, n (%)			
Left	12 (52)	13 (65)	0.395^b^
Right	11 (48)	7 (35)	
Classification, n(%)Cerebral infarction	21 (91)	20 (100)	
			0.532^b^
Cerebral hemorrhage	2 (9)	0 (0)	

n, sample size; y, years; d, day; IQR, Interquartile Range; Values shown are n (%) for categorical variables and median (IQR) for continuous variables that were not comply with a normal distribution; ^a^ Performed by Mann–Whitney test; ^b^ Performed by χ^2^ test.

### Primary outcome measure

The TIS, as the primary outcome measure, was showed a significant improvement in the experimental group compared with the control group after 3 weeks treatment [4 (2, 5) vs. 3 (1.25, 4), *p* = 0.030] (Table 2).

**Table 2:** Comparison of TIS, FMA, FMA-UE, FMA-LE, BBS, MBI.

Outcome measure	T_0_		T_1_		T_1_-T_0_		value of *p*
	Experimental group (*n* = 23)	Control group (*n* = 20)	Experimental group (*n* = 23)	Control group (*n* = 20)	Experimental group (*n* = 23)	Control Group (*n* = 20)	
TIS	14 (13, 16)	13.5 (12.25, 15.75)	18 (17, 20)	16.5 (15.25, 19)	4 (2, 5)	3 (1.25, 4)	0.030
FMA	50 (39, 65)	45.5 (24.25, 80.25)	63 (49, 81)	51 (25.75, 81)	14 (7, 21)	2 (0.25, 3.75)	0.001
FMA-UE	27 (18, 38)	23 (6.25, 54.75)	40 (28, 52)	27.5 (6.75, 56)	10 (6, 18)	1 (0, 3)	0.002
FMA-LE	23 (22, 27)	22 (18, 26)	26 (23, 30)	24 (18.50, 27.75)	2 (1, 4)	1 (0, 2)	0.009
BBS	36 (30, 46)	37 (25, 42.75)	47 (42, 51)	42.5 (30.50, 48.75)	5 (3, 14)	4 (1.25, 6)	0.221
MBI	60 (55, 70)	55 (46.25, 60)	75 (60, 80)	60 (55, 78.75)	10 (0, 25)	10 (5, 18.75)	0.814
Change of IAP (kPa)	16.33 (10.33, 20.67)	12.33 (9, 17.08)	11.33 (7.60, 14.67)	15.5 (8.83, 18.25)	−3 (−8, −1.33)	−0.02 (−3.08, 6)	0.011

T_0_, baseline data; T_1_, data of 3th weeks after baseline; T_1_-T_0_, the difference before and after intervention; TIS, The trunk impairment scale; FMA, The Fugl-Meyer assessment; FMA-UE, The Fugl-Meyer assessment- upper extremity; FMA-LE, The Fugl-Meyer assessment- lower extremity; BBS, Berg Balance Scale; MBI, The modified Barthel Index; IAP, Intra-abdominal pressure; P, comparison of difference between groups before and after intervention.

### Secondary outcome measures

Comparisons of some secondary outcome measures between two groups showed a significant improvement after 3-weeks treatment (Table 2). The experimental group outperformed the control group in terms of the change in IAP [−3 (−8, −1.33) vs. −0.02 (−3.08, 6), *p* = 0.011], FMA-UE [10 (6, 18) vs. 1 (0, 3), *p* = 0.002], FMA-LE [2 (1, 4) vs. 1 (0, 2), *p* = 0.009] and FMA [14 (7, 21) vs. 2 (0.25, 3.75), *p* = 0.001]. There was no significant difference between BBS [5 (3, 14) vs. 4 (1.25, 6), *p* = 0.221] and MBI [10 (0, 25) vs. 10 (5, 18.75), *p* = 0.814] between the two groups.

Comparisons of static balance ability were shown in Table 3. No significant differences were found in COP trajectory [−6 (−37.17, 1.17) vs. −12.67 (−48.17, 0.38), *p* = 0.297] or area [−3 (−22.67, 1.67) vs. −7.67 (−35.75, −1.58), *p* = 0.183] in the sitting posture with eyes open and no substantial changes in COP trajectory [−7.67 (−22.33, 2) vs. −10.67 (−38.33, 0.67), *p* = 0.481] or area [−1.33 (−9.83, 4.5) vs. −5.67 (−23.75, 0.08), *p* = 0.062] with eyes closed. With the patient’s eyes open in the upright posture, neither COP trajectory [−70.67 (−151, 38.71) vs. −42.33 (−153.67, 17.33), *p* = 0.817] nor area [−93.17 (−375.38, 27.08) vs. − 38 (−200.83, 276), *p* = 0.198] exhibited a significant difference. Similarly, with closed eyes, neither COP trajectory [−96.67 (−180.50, −27.83) vs. −51.33 (−193.67, 55), *p* = 0.424] nor area [−153.84 (−593.80, −55.5) vs. −160 (−297.67, 86.17), *p* = 0.355] were found to be significantly different.

**Table 3:** Comparison of static balance ability.

Outcome measure	T_0_		T_1_		T_1_-T_0_		*p*-value
	Experimental group (*n* = 21)	Control group (*n* = 20)	Experimental group (*n* = 21)	Control group (*n* = 20)	Experimental group (*n* = 21)	Control group (*n* = 20)	
Static open eye sitting balance test							
COP area (mm^2^)	12 (6.67, 36.83)	18.33 (8, 65.17)	9.67 (4.67, 15.33)	7.17 (4.84, 13.17)	−3 (−22.67, 1.67)	−7.67 (−35.75, −1.58)	0.183
COP trajectory (mm)	85.33 (61.67, 103.67)	102.33 (71.67, 129.58)	71 (61.33, 79)	77.17 (61.17, 107)	−6 (−37.17, 1.17)	−12.67 (−48.17, 0.38)	0.297
Static closed eye sitting balance test							
COP area (mm^2^)	7.33 (5.17, 16)	12.67 (6.50, 41.67)	7.33 (4.33, 14.67)	7 (5.17, 18.67)	−1.33 (−9.83, 4.5)	−5.67 (−23.75, 0.08)	0.062
COP trajectory(mm)	81 (64.17, 94.33)	94.17 (75.42, 128.33)	69 (63.17, 77)	76.67 (67.17, 95.25)	−7.67 (−22.33, 2)	−10.67 (−38.33, 0.67)	0.481
	Experimental group (*n* = 18)	Control group (*n* = 17)	Experimental group (*n* = 18)	Control group (*n* = 17)	Control group (*n* = 18)	Control group (*n* = 17)	
Static open eye standing balance test							
COP area (mm^2^)	259.42 (123.50, 625.88)	256 (111, 467)	165.67 (67.92, 282.83)	250 (127.83, 550.83)	−93.17 (−375.38, 27.08)	−38 (−200.83, 276)	0.198
COP trajectory(mm)	276.67 (217.25, 363)	374 (256.17, 476.33)	252.33 (156.33, 381.08)	308 (234.33, 445.67)	−70.67 (−151, 38.71)	−42.33 (−153.67, 17.33)	0.817
Static closed eye standing balance test							
COP area (mm^2^)	711 (351.33, 1468.08)	418.33 (293.17, 810.17)	463.83 (173.83, 1030.92)	416 (212.17, 776.33)	−153.84 (−593.80, −55.5)	−160 (−297.67, 86.17)	0.355
COP trajectory (mm)	473.50 (398.50, 602.75)	428 (337.67, 628.33)	413.50 (263.92, 699.92)	399 (357.50, 486)	−96.67 (−180.50, −27.83)	−51.33 (−193.67, 55)	0.424

T_0_, baseline data; T_1_, data of 3th weeks after baseline; T_1_-T_0_, the difference before and after intervention; COP, center of pressure; P, comparison of difference between groups before and after intervention.

Comparisons of lung function indexes were shown in Table 4. No significant differences were found in changes of MIP [5.4 (−2, 12.13) vs. −2 (−9.18, 10), *p* = 0.211], MEP [−0.25 (−11.13, 7.6) vs. 0.5 (−13.18, 11.08), *p* = 0.910], FVC [6.85 (−3.53, 20.48) vs. 1.9 (−6.85, 12.4), *p* = 0.192], FEV1 [3.95 (−4.9, 15.33) vs. −4.15 (−9.63, 5.38), *p* = 0.146], PEF [4.7 (−5.05, 10.43) vs. 0.15 (−8.93, 7.08), *p* = 0.446] or MMEF [−6.3 (−18.38, 0.95) vs. 0.95 (−11.55, 25.78), *p* = 0.211] between two groups. The FVC in the experimental group was significantly improved compared to baseline [81.35 (63.30, 94.88) vs. 91.75 (79.40, 97.90), *p* = 0.02].

**Table 4:** Comparison of lung function indexes.

Outcome measure	T_0_		T_1_		T_1_-T_0_		*p*-value
	Experimental group (*n* = 22)	Control group (*n* = 18)	Experimental group (*n* = 22)	Control group (*n* = 18)	Experimental group (*n* = 22)	Control group (*n* = 18)	
MIP (%pred)	36.80 (29.23, 49.08)	44.80 (27.85, 63.58)	41.50 (30.85, 55.43)	42.10 (31.63, 61.78)	5.4 (−2, 12.13)	−2 (−9.18, 10)	0.211
MEP (%pred)	57.10 (38.33, 66.15)	41.65 (33.50, 66.70)	51.55 (35.18, 73.48)	49.20 (34.65, 60.73)	−0.25 (−11.13, 7.6)	0.5 (−13.18, 11.08)	0.910
FVC (%pred)	81.35 (63.30, 94.88)	79.25 (48.48, 104.53)	91.75 (79.40, 97.90) ^*^	85.55 (54.45, 101.83)	6.85 (−3.53, 20.48)	1.9 (−6.85, 12.4)	0.192
FEV1 (%pred)	78.05 (62, 89.23)	79.50 (59.73, 102.50)	83.70 (72.80, 98.55)	81.20 (66.76, 103.88)	3.95 (−4.9, 15.33)	−4.15 (−9.63, 5.38)	0.146
PEF (%pred)	48.20 (32.33, 60)	44.50 (28.78, 58.30)	48.25 (37.05, 66.03)	44.25 (33.05, 56.58)	4.7 (−5.05, 10.43)	0.15 (−8.93, 7.08)	0.446
MMEF (%pred)	59.05 (48.95, 75.80)	69.40 (39.90, 87.13)	53.25 (37.85, 73.85)	70.85 (44.33, 89.53)	−6.3 (−18.38, 0.95)	0.95 (−11.55, 25.78)	0.211

T_0_, baseline data; T_1_, data of 3th weeks after baseline; T_1_-T_0_, the difference before and after intervention; MIP, maximum inspiratory pressure; MEP, maximum expiratory pressure; FVC, forced vital capacity; FEV1, forced expiratory volume in the first second; PEF, peak expiratory flow; MMEF, maximum expiratory mid-flow; P, comparison of difference between groups before and after intervention; *p-value < 0.05, intra-group comparison before and after intervention, *p* = 0.02.

## Discussion

In the current study, we compared two different therapies, WAT and TSAT, to assess their clinical effects of the trunk postural control disorder. The results showed that patients with trunk deficits treated with WAT achieved better trunk postural control, motor function and the FVC than TSAT, which supported our hypothesis.

### Trunk postural control disorder of stroke

Most of the published literature pays most attention to hemiplegic limb rehabilitation after stroke while neglecting the importance of trunk recovery (Karthikbabu et al., 2011). According to Devis, postural control disorder is related to a deterioration in upper and lower limb functions, balance, position shift, walking, breath and speech (Jamal et al., 2018). The weakness of the abdominal muscles and the spasticity of the extensor of the trunk make the hemiplegic side trunk adopt an abnormal and frozen mode of movement. This movement is known as the synergic movement pattern and is characterized by the loss of selective movement. Regardless of the position of the hemiplegic patient, the loss of selective activities among trunk muscles or between the trunk and the limb exists, which hinders a patient’s ability to bend forward, bend laterally, extend against gravity, rotate in multiple dimensions, and maintain or transfer their center of gravity (COG). Therefore, strengthening weak abdominal muscles and alleviating trunk spasticity are the key to restoring trunk stability and flexibility, which will ultimately promote selective trunk activities.

### WAT improves trunk postural control

The front and center line of the human body is Ren channel, the sea of Yin pulse, meaning blood; the posterior median line is the Du channel, which acts as the Yang vein of the whole body and mains qi. Therefore, Tai Chi attaches great importance to trunk movement, which is conducive to keep the two veins of Ren and Du open and the balance of Yin and Yang. Moreover, Tai Chi emphasizes the spiral circular motion track of the trunk and limbs, so that the joints, muscles and ligaments of the whole body can achieve the effect of Qi and blood running and activating collaterals through the uniform and coherent repeated activities, which is beneficial to the functional recovery of stroke patients with hemiplegia.

The concept of “Taking the Waist as the Axis” originates from Tai Chi, which emphasizes various arc-shaped track movements of the waist, e.g., axial rotation, composite rotation and diagonal rotation, from a small to a wide range, thus, allowing the waist to achieve flexibility. It should be noted that various trunk rotations are involved in daily activities, implying that the treatment based on trunk rotation may be more in keeping with the demands of a stroke patient’s daily life.

WAT that involves multidimensional trunk rotations is mainly performed by the bilateral obliquus internus abdominis (OI) and obliquus externus abdominis (OE) muscles (Urquhart and Hodges, 2005). The ipsilateral OI and contralateral OE act as dynamic muscles for centripetal contraction, whereas ipsilateral OE and contralateral OI function as fixed anchors for centrifugal contraction (Ng et al., 2001). “Ye Ma Fen Zong” (野马分鬃, Figure 1B) comprises axial rotations at 45° and 20° degrees alternately and repeatedly to activate the bilateral OE and OI, thus increasing trunk flexibility. Furthermore, trunk rotation activates the transversus abdominis, which is associated with trunk stability (Urquhart and Hodges, 2005), and IAP is a sensitive indicator of trunk stability. Therefore, WAT exhibited a more stable IAP than TSAT.

The abdominal muscles show much higher co-activation than the back muscles during lateral flexion (Huang et al., 2001). Due to the lack of efficient abdominal contractions, the trunk lateral flexion is substituted by overall trunk flexion with excessive abduction of the non-hemiplegic upper limb. During TSAT, the therapist provides considerable assistance to help the patient complete the movements of lateral flexion. In contrast, the composite trunk rotation of the “Zuo You Xia Shi” (左右下势, Figure 1G) and the diagonal rotation of the “Hai Di Zhen” (海底针, Figure 1F), whose processes involve combinations of lateral flexion, forward flexion and anti-gravity stretching, can be easily performed by controlling the patient’s shoulders, or managing the manubrium and thoracic vertebrae. Consequently, the buckling capacity of the lower trunk is improved. The synergistic movement patterns of the trunk and lower limbs are effectively inhibited by trunk multidimensional rotation, with the abduction of the lower limbs in the sitting position. During “Lan Que. Wei” (揽雀尾, Figure 1D), the trunk rotates from the front to the posterolateral space, and then the upper trunk pulls the COG to the opposite side, challenging the patient’s dynamic control of upper trunk.

It is well-known that trunk rotation is the pivotal factor in reducing hypertonicity (Davies, 1990). WAT stretches the spastic muscles of the trunk through various trunk rotation training, especially in a slow, continuous, relaxed and repetitive movement rhythm that is unique to Tai Chi. From the initial passive trunk rotation to the active trunk rotation guided by controlling the scapula, thoracic vertebra or pelvis, the compensatory posture and spasms of the trunk have been progressively suppressed. For instance, “Dao Juan Gong” (倒卷肱, Figure 1E) maybe relieve trunk spasticity by assisting patient to rotate the trunk to the one side and then extend the contralateral leg to extend backward, and pause for several seconds. “Hai Di Zhen” (海底针, Figure 1F) maybe relieve trunk spasticity by guiding the hemiplegic trunk and arm to extend in the direction of the contralateral toe. Finally, the patient is able to complete various trunk rotation independently in sitting or standing position.

TIS employed in this study was to evaluate the capacity of maintaining trunk stability and performing selective trunk movements (Verheyden et al., 2009; Alhwoaimel et al., 2018). WAT relieves trunk spasm by slowly rotating the trunk, promotes trunk stability by activating abdominal muscles, and improves selective trunk control by multi-dimensional rotation, thus improving TIS scores. In the present trial, it was found that WAT had a superior impact on improving trunk postural control compared to TSAT for hemiplegic patients.

### Improvement of limb functions

Upper limb mobility relies on the shoulder girdle’s proximal fixation, which in turn depends on thorax stability, which is achieved by contraction of the abdominal muscles to keep the ribs in a descending position. WAT, based on the trunk rotation, strengthens the rectus abdominis and external abdominal oblique muscles, counteracting excessive upward and lateral movement of the thorax, thus stabilizing the shoulder and improving upper limb performance. Surprisingly, the present trail results found superior limb function recovery after WAT compared to TSAT. Hemiplegic upper limbs often abduct with an abnormal flexion synergy pattern which makes them unable to engage in a reaching function (Ellis et al., 2017). Additionally, hemiplegic patients have difficulty with flexion and abduction of the shoulder, which is accompanied by elevation of the hemiplegic shoulder girdle, leading to subacromial impingement and shoulder pain.

Previous studies have demonstrated the “Loud Hand” of Tai Chi significantly improved the limb motor function, with strengthening the internal and external rotation of the shoulder, promoting forward flexion and abduction of the shoulder and alleviating shoulder pain (Lyu et al., 2018; Luo et al., 2020; You et al., 2021). WAT applies round rotation motion for both trunk and limb training. In the “Lou Xi Ao Bu” (搂膝拗步, Figure 1C), the unilateral shoulder is rotated outward and which is then extended forward with the palm pushed forward. Simultaneously, the contralateral shoulder is rotated inward and then returned to the neutral position with the palm pressed down, which can effectively prevent shoulder injury and also promote shoulder function. The slow rotation of the shoulder relieves the high tension of the internal rotators induced by hemiplegia, whilst the up-down movement of the upper limbs prevents spinal overextension and facilitates isolated movement between the upper limbs. Anterior–posterior lunge training and trunk rotations are performed simultaneously in a standing position, which may promote isolated movement between the trunk and lower limbs.

Thus, satisfactory trunk function plays an essential role in accelerating limb functions. The isolated movement of the affected limbs is driven by the trunk rotation movement, which is consistent with the concept of “Taking the Waist as the Axis,” that is, the trunk leads the entire body.

### Balance function

WAT involves multi-directional weight shifts, such as the “Zuo You Xia Shi” (左右下势, Figure 1G) with the COG moving left, right, up and down. Also, the “Hai Di Zhen” (海底针, Figure 1F) with COG moving back and forth in a diagonal orientation and “Zhuan Shen Ban Lan Chui” (转身搬拦捶, Figure 1H) with COG shifting between left and right. Furthermore, the slow, continuous, relaxed and repetitive movement patterns enhance proprioceptive input. Therefore, after the WAT intervention, the experimental group obtained a trend of improvement in the COP trajectory with eyes closed in sitting position. Unfortunately, given the short intervention period, we could not detect differences in measures related to balance between the two groups. As daily living ability was strongly associated with balance, there was no significant difference in MBI scores between the two groups accordingly (Kim and Park, 2014).

### Lung function

A reduced respiratory function is frequently found in hemiplegic patients, usually characterized by respiratory muscle weakness and altered chest wall kinematics (Lima et al., 2014; Menezes et al., 2016). During WAT, the thorax is repeatedly deformed and restored with repetitive trunk rotation training, which benefits flexibility of the thorax. Thus, the improved abdominal muscles help draw the rib cage down, promoting the expansion of the thorax symmetrically, thus improving lung ventilation. The within-group differences in FVC following WAT might be attributed to the above findings. However, there was no difference in FVC between the two groups that might relate to a 3-week intervention that was insufficient.

## Limitations

This study had a number of limitations. The intervention only lasted for 3 weeks, and no follow-ups were conducted, making it impossible to assess the long-term influence of the therapy regiments. Besides, the results still need to be interpreted with caution for the measurement we used mostly are the scales. Further randomized studies with a larger cohort size and longer duration of study will be required to confirm its clinically worthwhile benefits. In addition, more quantitative measures should be emphasized in the future research. What’s more, additional studies should be well-designed to implement WAT in stroke patients with varying degrees of trunk dysfunction to explore the correlation between the trunk and limb functions.

## Conclusion

Compared to “Trunk Selective Activity” therapy (TSAT), it was found that “Taking the Waist as Axis” therapy (WAT) not only improved trunk postural control in stroke patients with hemiplegia but also produced significant improvements in their motor functions. Besides, within group, WAT showed an improvement in forced vital capacity (FVC). The present clinical trial supports the feasibility and effectiveness of WAT derived from Tai Chi for the treatment of stroke patients and provides an important reference for employing traditional fitness regimes in stroke rehabilitation.

## Data availability statement

The raw data supporting the conclusions of this article will be made available by the authors, without undue reservation.

## Ethics statement

The studies involving human participants were reviewed and approved by the Shanghai Xuhui Central Hospital Ethics Committee. The patients/participants provided their written informed consent to participate in this study. Written informed consent was obtained from the individual(s) for the publication of any potentially identifiable images or data included in this article.

## Author contributions

RC and JW: formal analysis. RC, YingZ, and ML: writing – original draft. YingZ and WF: writing – review, revise and finalize the manuscript. LY, YanW, YanmW, and YiZ: patient recruitment and enrollment. JM, GL, and PH: assessment. JY and HL: methodology – “Taking the Waist as Axis” therapy. ML and WN: methodology – “Trunk Selective Activity” therapy. FW and JP: methodology – conventional therapy. CC and ZP: data curation. NZ: financial management. All authors contributed to the article and approved the submitted version.

## Funding

This study was funded by 2021 Shanghai Sports Science and Technology National Fitness Program Project (No. 21Q001), 2020 Shanghai Xuhui district scientific research project (No. SHXH202032), and 2021 Shanghai General Hospital Integrated Chinese and Western Medicine Special Project (No. ZHYY-ZXY JHZX-202109).

## Conflict of interest

The authors declare that the research was conducted in the absence of any commercial or financial relationships that could be construed as a potential conflict of interest.

## Publisher’s note

All claims expressed in this article are solely those of the authors and do not necessarily represent those of their affiliated organizations, or those of the publisher, the editors and the reviewers. Any product that may be evaluated in this article, or claim that may be made by its manufacturer, is not guaranteed or endorsed by the publisher.

## Abbreviations

WAT: “Taking the Waist as the Axis” Therapy
TSAT: “Trunk Selective Activity” Therapy
TIS: Trunk Impairment Scale
FMA: Fugl-Meyer Assessment
BBS: Berg Balance Scale
MBI: Modified Barthel Index
ADL: activities of daily living
MMSE: A Mini-Mental State Examination
IAP: intra-abdominal pressure
IQR: interquartile range
COP: central of pressure
MIP: maximum inspiratory pressure
MEP: maximum expiratory pressure
FEV1: forced expiratory volume in the first second
FVC: forced vital capacity
PEF: peak expiratory flow
MMEF: maximum expiratory mid-flow
%pred: percentage of the predicted values
TrA: transverse abdominis
COG: center of gravity.
